# Supplementary material for: Lipid Profile, Lipoprotein Subfractions, and Fluidity of Membranes in Children and Adolescents with Depressive Disorder: Effect of Omega-3 Fatty Acids in a Double-Blind Randomized Controlled Study
Source: Biomolecules. 2020 Oct 8;10(10):1427. doi: 10.3390/biom10101427 (PMC7650679; doi:10.3390/biom10101427)
Supplement: Supplementary file 1 [file biomolecules-10-01427-s001.zip › Supplement Figure S1.pdf]

CONSORT

TRANSPARENT REPORTING of TRIALS

CONSORT 2010 Flow Diagram

Enrollment

Assessed for eligibility (n=96)

Excluded (n=36)
- Not meeting inclusion criteria (n=11)
- Declined to participate (n=10)
- Travelling problems (n=6)
- Intolerance to blood taking (n=9)

Randomized (n=60)

Allocation

Omega 3 (n= 30)
- Received allocated intervention (n= 30)
- Did not receive intervention whole period (n=1)
  Taste of supplement (n=1)
- Subgroup with DD (n=17, 58.6%)
- Subgroup with MADD (n=12, 41.4%)
- Treated for at least one month before enrollment (n=14. 48.3%)
- Firstly diagnosed (n=15, 51.7%)

Omega 6 (n= 30)
- Received allocated intervention (n=30)
- Did not receive intervention whole period (n=1)
  Non-compliance (n=1)
- Subgroup with DD (n=13, 44.8%)
- Subgroup with MADD (n=16, 55.2%)
- Treated for at least one month before enrollment (n=10, 34.5%)
- Firstly diagnosed (n=19, 65,5%)

Follow-Up

Discontinued participation after week 12 (n=1)
- Non-compliance (n=1)

Discontinued participation after week 12 (n=5)
- Non-compliance (n=1)
- Problem with travelling (n=2)
- Intolerance to blood taking (n=2)

Investigation

Analysed supplemented (n=29)
- Excluded from analysis (n=0)
- Treated with antidepressants during intervention (n=24)
- No treatment during intervention (n=5)

Analysed supplemented (n=29)
- Excluded from analysis (n=0)
- Treated with antidepressants during intervention (n=22)
- No treatment during intervention (n=7)
