# Supplementary material for: Lipid Profile, Lipoprotein Subfractions, and Fluidity of Membranes in Children and Adolescents with Depressive Disorder: Effect of Omega-3 Fatty Acids in a Double-Blind Randomized Controlled Study
Source: Biomolecules. 2020 Oct 8;10(10):1427. doi: 10.3390/biom10101427 (PMC7650679; doi:10.3390/biom10101427)
Supplement: Supplementary file 1 [file biomolecules-10-01427-s001.zip › Supplement S3.pdf]

Supplement S3. LDL subfractions in serum of controls and patients at the baseline and durin the intervention at the week 6 and 12

	Controls			All		Om3						Om6						p Om3 vs Om6		
Subfraction		±SD	p C vs P	Week 0	±SD	Week 6	±SD	p 0 vs 6	Week 12	±SD	p 0 vs 12	Week 6	±SD	p 0 vs 6	Week 12	±SD	p 0 vs 12	Week 0	Week 6	Week 12
(mg/mL)	20			60		29			26			28			26					
VLDL	19,3	4,5	0,001	26,1	8,1	25,2	7,7	0,328	24,6	9,4	0,134	25,5	7,3	0,724	23,3	7,7	0,056	0,531	0,974	0,345
IDL-1	22,0	3,3	0,973	21,9	13,0	21,4	5,6	0,125	20,6	5,9	0,560	21,8	6,4	0,480	21,0	6,0	0,366	0,346	0,706	0,775
IDL-2	7,8	3,4	0,015	6,0	2,6	6,5	3,3	0,480	6,7	3,0	0,286	6,1	2,2	0,475	6,6	2,4	0,012	0,409	0,298	0,712
IDL-3	13,9	3,6	0,432	12,9	5,3	12,5	6,2	0,508	12,6	4,4	0,838	12,6	5,3	0,886	13,2	4,9	0,438	0,391	0,859	0,528
LDL-1	36,8	10,2	0,028	31,0	10,1	35,0	11,2	0,001	34,6	12,2	0,006	34,1	11,2	0,032	34,4	10,5	0,115	0,639	0,383	0,615
LDL-2	9,4	7,4	0,296	7,7	6,1	10,7	8,7	0,003	8,1	4,3	0,135	11,3	8,7	0,049	10,9	8,2	0,235	0,607	0,746	0,643
LDL-3	0,0	0,0	0,137	0,2	0,7	0,6	1,3	0,110	0,3	0,9	0,600	0,9	3,5	0,264	0,5	1,1	0,312	0,822	0,772	0,869
LDL-4	0,0	0,0		0,0	0,0	0,0	0,0		0,0	0,2	0,327	0,1	0,4	0,326	0,0	0,2	0,327		0,303	0,323
LDL-5	0,0	0,0		0,0	0,0	0,0	0,0		0,0	0,0		0,0	0,0		0,0	0,0				
LDL-6	0,0	0,0		0,0	0,0	0,0	0,0		0,0	0,0		0,0	0,0		0,0	0,0				
LDL-7	0,0	0,0		0,0	0,0	0,0	0,0		0,0	0,0		0,0	0,0		0,0	0,0				

VLDL - very low density lipoproteins, IDL - intermediate density lipoproteins, LDL - low density lipoproteins, C - controls, P - patients, Om3 - omega-3 group, Om6 - omega-6 group, n - number of subjects, SD - standard deviation, p - significance
