# Supplementary material for: Lipid Profile, Lipoprotein Subfractions, and Fluidity of Membranes in Children and Adolescents with Depressive Disorder: Effect of Omega-3 Fatty Acids in a Double-Blind Randomized Controlled Study
Source: Biomolecules. 2020 Oct 8;10(10):1427. doi: 10.3390/biom10101427 (PMC7650679; doi:10.3390/biom10101427)
Supplement: Supplementary file 1 [file biomolecules-10-01427-s001.zip › Supplement S4.pdf]

Supplement S4. HDL subfractions in serum of controls and patients at the baseline and durin the intervention at the week 6 and 12

	Controls			All		Om3						Om6						p Om3 vs Om6		
Subfraction		±SD	p C vs P	Week 0	±SD	Week 6	±SD	p 0 vs 6	Week 12	±SD	p 0 vs 12	Week 6	±SD	p 0 vs 6	Week 12	±SD	p 0 vs 12	Week 0	Week 6	Week 12
(% Area)	n=21			n=58		n=28			n=26			n=29			n=27					
L-HDL1	7,1	5,5	0,006	10,6	4,4	12,9	4,7	0,000	12,6	5,1	0,006	10,9	3,6	0,904	12,1	6,3	0,390	0.657	0,084	0,754
L-HDL2	17,0	3,9	0,745	16,6	4,0	19,3	3,5	0,001	18,4	5,1	0,052	16,8	3,7	0,671	15,5	5,2	0,548	0.541	0,011	0,046
L-HDL3	12,0	2,6	0,588	11,7	2,4	13,9	2,9	0,021	13,2	3,5	0,025	12,4	3,3	0,370	11,5	3,2	0,924	0.396	0,070	0,061
L-HDL1-3	36,0	10,1	0,235	38,8	9,0	44,4	7,3	0,020	44,0	10,5	0,003	39,6	8,1	0,731	38,6	12,1	0,851	0.878	0,025	0,097
Chol 1-3 mg/mL	15,4	6,3	0,022	20,1	8,4	23,9	9,6	0,001	20,2	8,8	0,201	21,5	8,4	0,696	20,6	9,6	0,904	0.489	0,340	0,887
I-HDL4	13,6	2,0	0,031	12,5	1,8	13,4	1,8	0,004	13,3	3,1	0,052	13,1	2,3	0,400	12,9	3,2	0,384	0.751	0,489	0,600
I-HDL5	13,1	2,5	0,030	11,9	1,9	11,7	2,6	0,105	12,2	1,7	0,848	11,4	2,0	0,650	11,3	2,8	0,705	0.150	0,631	0,168
I-HDL6	20,2	3,6	0,743	20,6	4,3	18,2	4,3	0,000	19,0	5,3	0,013	19,6	3,8	0,537	19,8	5,1	0,403	0.495	0,201	0,569
I-HDL7	5,4	1,4	0,166	6,0	1,7	5,1	1,6	0,002	5,4	2,2	0,007	5,7	1,6	0,549	5,9	1,9	0,502	0.879	0,158	0,374
I-HDL4-7	53,2	5,8	0,157	51,1	6,0	48,3	6,8	0,001	50,0	7,6	0,220	49,8	5,5	0,650	50,0	9,0	0,686	0.381	0,373	0,987
Chol 4-7 mg/mL	21,8	3,1	0,007	25,3	5,4	25,3	5,8	0,959	24,8	7,5	1,000	26,0	4,9	0,543	25,7	6,9	0,976	0.996	0,630	0,654
S-HDL8	4,2	1,8	0,261	4,7	1,8	4,3	1,7	0,155	4,2	2,2	0,012	5,0	1,7	0,463	5,3	2,1	0,257	0,786	0,128	0,078
S-HDL9	2,9	1,9	0,578	3,1	1,5	2,6	1,5	0,119	2,7	1,8	0,103	3,1	1,6	0,990	3,4	2,0	0,676	0,802	0,153	0,158
S-HDL10	3,5	3,7	0,089	2,2	2,7	1,5	2,4	0,290	1,4	2,1	0,060	2,1	3,5	0,995	2,4	3,0	0,703	0,916	0,432	0,173
S-HDL8-10	10,4	7,1	0,805	10,0	5,2	8,3	4,9	0,110	8,2	5,5	0,011	10,4	5,7	0,844	11,1	6,6	0,618	0,922	0,144	0,092
Chol 8-10 mg/mL	11,7	3,2	0,356	11,1	3,0	8,6	3,0	0,299	8,9	3,3	0,243	10,9	3,3	0,766	11,4	3,7	0,622	0,966	0,238	0,099

HDL - density lipoproteins, L-HDL - large HDL lipoprteins, I-HDL - intermediate HDL lipoproteins, S-HDL - small HDL lipoproteins, Cho - cholesterol, C - controls, P - patients, Om3 - omega-3 group, Om6 - omega-6 group, n - number of subjects, SD - standard deviation, p - significance
