# Supplementary material for: Lipid Profile, Lipoprotein Subfractions, and Fluidity of Membranes in Children and Adolescents with Depressive Disorder: Effect of Omega-3 Fatty Acids in a Double-Blind Randomized Controlled Study
Source: Biomolecules. 2020 Oct 8;10(10):1427. doi: 10.3390/biom10101427 (PMC7650679; doi:10.3390/biom10101427)
Supplement: Supplementary file 1 [file biomolecules-10-01427-s001.zip › Supplement S5.pdf]

Supplement S5. Activities of PON-L and PON-A in controls and in patients at the baseline and during the intervention at the week 6 and week 12

PON (U/mL)	Controls			All		Om3						Om6					
		±SD	p C vs P	Week 0	±SD	Week 6	±SD	p 0 vs 6	Week 12	±SD	p 0 vs 12	Week 6	±SD	p 0 vs 6	Week 12	±SD	p 0 vs 12
	n=20			n=59		n=29			n=26			n=28			n=26		
PON-L	10.6	2.6	0.520	10.2	2.6	10.9	3.5	0.084	9.6	2.2	0.146	10.7	3.8	0.997	11.1	3.7	0.919
PON-A	122.7	23.0	0.142	117.2	26.1	115.7	30.2	0.249	118.5	29.3	0.519	115.4	20.6	0.805	119.9	20.4	0.352

PON - paraoxonase 1, PON-L - lactonase activity of PON 1, PON-A - arylesterase activity of PON 1, Om3 - omega-3 group, Om6 - omega-6 group, SD - standard deviation, V - controls, P - patients, p - significance, n - number of subjects
