# Supplementary material for: Lipid Profile, Lipoprotein Subfractions, and Fluidity of Membranes in Children and Adolescents with Depressive Disorder: Effect of Omega-3 Fatty Acids in a Double-Blind Randomized Controlled Study
Source: Biomolecules. 2020 Oct 8;10(10):1427. doi: 10.3390/biom10101427 (PMC7650679; doi:10.3390/biom10101427)
Supplement: Supplementary file 1 [file biomolecules-10-01427-s001.zip › Supplement Table S1.pdf]

Supplement Table S1. Biochemical martkers in controls and patients at the baseline and durin the intervention at the week 6 and 12

		Physiol.	Controls			All		Om3						Om6					
Marker	Unit	values		±SD	p C vs P	Week 0	±SD	Week 6	±SD	p 0 vs 6	Week 12	±SD	p 0 vs 12	Week 6	±SD	p 0 vs 6	Week 12	±SD	p 0 vs 12
		n	20			60		28			25			25			26		
Glu	mmol/L	4.2-6.1	4.89	0.5	0.66	4.79	0.5	4.78	0.4	0.684	4.65	0.6	0.791	4.95	0.8	0.385	5.07	0.91	0.138
CreV	µmol/L	21-85	59.65	12.4	0.22	63.04	11.0	62.64	11.1	0.806	64.12	12.3	0.744	62.04	10.2	0.627	62.38	9.60	0.725
UA	µmol/L	100-360	296.65	70.9	0.23	258.45	73.7	276.21	79.4	0.883	271.92	72.7	0.852	268.63	49.9	0.753	268.62	56.17	0.761
TCH	mmol/L	3.87-5.2	3.85	0.6	0.718	3.94	0.6	4.21	0.7	0.051	4.04	0.8	0.038	4.18	0.8	0.082	4.18	0.68	0.079
HDL	mmol/L	>0.9	1.41	0.3	0.222	1.54	0.3	1.61	0.4	0.171	1.53	0.3	0.208	1.66	0.4	0.041	1.62	0.39	0.141
LDL	mmol/L	<3.4	2.16	0.6	0.696	2.11	0.6	2.36	0.8	0.079	2.20	0.6	0.022	2.32	0.7	0.141	2.31	0.71	0.157
TAG	mmol/L	0-1.3	0.81	0.3	0.169	0.94	0.5	0.86	0.3	0.287	0.82	0.4	0.105	0.81	0.3	0.141	0.93	0.50	0.718
AI	LDL/HDL	<3.5	1.61	0.6	0.386	1.45	0.6	1.61	0.8	0.405	1.51	0.6	0.164	1.49	0.6	0.949	1.56	0.71	0.582
AST	µkat/L	0-0.85	0.39	0.1	0.693	0.38	0.1	0.43	0.1	0.066	0.37	0.1	0.173	0.39	0.1	0.625	0.38	0.11	0.914
ALT	µkat/L	0-0.75	0.27	0.1	0.710	0.32	0.2	0.40	0.3	0.011	0.28	0.1	0.614	0.35	0.2	0.142	0.28	0.11	0.984
GMT	µkat/L	0.17-1.7	0.18	0.1	0.748	0.18	0.1	0.21	0.1	0.113	0.19	0.1	0.072	0.21	0.1	0.092	0.21	0.11	0.147
CREA_U	mmol/L	7.0-18.0	16.59	7.9	0.14	11.94	8.9	16.09	12.3	0.224	15.85	10.5	0.815	12.38	8.9	0.710	12.05	9.99	0.617
hsCRP	mg/L		<0.,5			0.74	1.4	0.67	1.6	0.795	0.42	1.2	0.380	0.84	1.4	0.449	0.47	0.90	0.749

Glu - fasting glucose, CreV - creatinive, UA - uric acid, TCH - total cholesterol, HDL - HDL-cholesterol, LDL - LDL-cholesterol, TAG - triacylglycerols, AI - atherogenic index (LDL/HDL), AST - Aspartate Aminotransferase, ALT - Alanine Aminotransferase, GMT - Gamma-glutamyltransferase, Crea_U - creatinine in urine, hsCRP - hight sensitive C-rective protein, C -control, P - patients, n - number os subjects, SD - standard deviation, Om3 - Omega-3 group, Om6 - Omega-6 group, vs - versus
